# CircMAT2B Induced by TEAD1 Aggravates the Warburg Effect and Tumorigenesis of Oral Squamous Cell Carcinoma through the miR-942-5p/HSPD1 Axis

**DOI:** 10.1155/2022/7574458

**Published:** 2022-08-16

**Authors:** Chen Zou, Dahong Huang, Haigang Wei, Siyuan Wu, Jing Song, Zhe Tang, Xia Li, Yilong Ai

**Affiliations:** Foshan Stomatological Hospital, School of Medicine, Foshan University, Foshan, Guangdong, China

## Abstract

Oral squamous cell carcinoma (OSCC) is one of the most lethal cancers worldwide. The high morbidity and mortality of OSCC are a great burden to global health-care systems. Therefore it is important to understand the underlying molecular mechanisms of OSCC initiation and progression. This study aimed to investigate the role of circMAT2B in OSCC progression and its molecular mechanisms. First, the expression and circularization of circMAT2B in OSCC cells were verified. Subsequently, knockdown of circMAT2B was shown to inhibit OSCC cell proliferation, migration, invasion, and the Warburg effect. Bioinformatics prediction, RNA-pull down, and luciferase reporter gene assays led to the identification of a novel TEAD1/circMAT2B/miR-942-5p/HSPD1 axis in OSCC progression. In conclusion, the novel TEAD1/circMAT2B/miR-942-5p/HSPD1 axis is a potential target for OSCC.

## 1. Introduction

Oral squamous cell carcinoma (OSCC) is one of the most lethal cancer types globally, accounting for about 3% of overall malignant tumors, and 95% of head and neck cancers [1–3]. OSCC has a poor 5-year survival rate because of diffuse metastasis and frequent recurrence, so less than 50% of patients diagnosed with OSCC survive more than one year [4, 5]. Despite advancements in diagnosis and treatments made in recent years, the high morbidity and mortality of OSCC still represent an enormous health burden worldwide, with OSCC patients having a poor quality of life. Therefore, it is important to understand the underlying molecular mechanisms of OSCC to develop more effective therapies.

Circular RNAs (circRNAs) are a class of non-coding RNAs and products of back-splicing events from an exon to a covalently closed loop. A few circRNAs have been reported in the nucleus and regulate gene transcription. However most circRNAs are located in the cytoplasm, indicating the biological function of circRNAs in cellular behaviors [6, 7]. Structurally, circRNAs have conserved, stable, tissue-specific, subcellular location and developmental stage-specific characteristics, suggesting a specific biological role in cellular behaviors [8]. Mechanically, circRNAs can interact with miRNAs and proteins and can be translated into proteins [9–13]. Biologically, circRNAs are involved in multiple biological or pathological events, especially in cancers [14–18]; therefore, there are promising therapeutic targets for various diseases and they demand further exploration.

Recently, emerging evidence revealed the crucial role of circRNAs in OSCC progression. Zhao et al. demonstrated the function of circUHRF1 in OSCC tumorigenesis and cellular behaviors [19]. Xia et al. found that circular RNA MMP9 modulates OSCC progression via stabilizing MMP9 mRNA expression [20]. Zhang et al. revealed a novel circMDM2/miR-532-3p/HK2 axis in OSCC progression regulating glycolysis [21]. Chen et al. found that circATRNL1 modulates OSCC cell susceptibility to irradiation [22]. Of interest, our group reported that circular RNA Methionine Adenosyltransferase 2B (circMAT2B; hsa_circ_0074854) is involved in the progression of hepatocellular carcinoma, colorectal cancer, and gastric cancer [23–25]. However, whether circMAT2B participates in OSCC development remains unclear.

In the current study, we aimed to investigate the role of circMAT2B in OSCC progression, showing that circMAT2B is highly expressed in OSCC cells. Furthermore, the dysregulation of circMAT2B in OSCC cells influences cell proliferation, migration, invasion, and Warburg effects. Also, a novel circMAT2B/miR-942-5p/HSPD1 axis in OSCC progression was identified and circMAT2B expression in OSCC cells was transcriptionally regulated by TEAD1. This study has partially demonstrated the role of the TEAD1/circMAT2B/miR-942-5p/HSPD1 axis in OSCC development and might provide new insight for OSCC diagnosis and therapeutic research.

## 2. Materials and Methods

### 2.1. Cell Culture and Transfection

OSCC cell lines (HOK, SCC-9, SCC-15, Cal-27, HSC-3, and HSC-6) were commercially procured from the American Tissue Culture Collection (Manassas, VA, USA) and cultured in Dulbecco's modified Eagle's medium (DMEM) with 10% fetal bovine serum (FBS) (Gibco, USA) in a 37°C-humidity environment with 5% CO_2_. All mimics, siRNAs, shRNAs, and vectors were synthesized and procured from GeneChem company (Shanghai, China). Two shRNAs targeting circ-MAT2B were synthesized and inserted into a pLV-EF1a-EGFP(2A)-Puro vector, followed by a lentivirus package. 6 *μ*g/ml polybrene (Sigma-Aldrich, MO, USA) was used to perform the transfections. The sequences are listed as follows: si-TEAD1: GGACATTCGTCAGATTTAT,si-NC5′-CCTCTCGTGAAACCTCCTT-3′; miR-942-5p mimic 5′-UCUUCUCUGUUUUGGCCAUGUG-3′; miR-942-5p inhibitor 5′-CACAUGGCCAAAACAGAGAAGA-3. Lipofectamine® 2000 reagent (Invitrogen) was used to perform all the transfections according to the manufacturer's instructions.

### 2.2. qRT-PCR

Total RNA from cells and tissues was extracted using TRIzol® reagent (Invitrogen) following the manufacturer's protocol and reverse transcribed using gDNAEraser (Perfect Real Time) (Takara). qPCR was performed using an SYBR-Green Master Mix (TaKaRa) on a CFX96 Real Time System C1000 Cycler (Bio-Rad) according to the manufacturer's protocol. The housekeeping gene GAPDH was used as an internal control. Primers were described as follows: circMAT2B: F: 5′-GATCACTGGCAGCAGAGGTT-3′, R: 5′-CAGTGGCACCAGTAACCAGA-3′, MAT2B: F: 5′-ACAGAGAGGAAGACATACCAG-3′, R: 5′-GTTCATTGCCAGACCAGTG-3′, TEAD1: F: 5′-GGACAGGCAAGACGAGGA-3′, R: 5′-AGTGGCCGAGACGATCTG-3′, miR-942-5p: F: 5′-AGGGTCTTCTCTGTTTTGGC-3′, R: 5′-GTTGTGGTTGGTTGGTTTGT-3′, HSPD1: F: 5′-AAATTGCACAGGTTGCTACG-3′, R: 5′-TGATGACACCCTTTCTTCCA-3′, GAPDH: F: 5′-GGGAAACTGTGGCGTGAT-3′, R: 5′-GAGTGGGTGTCGCTGTTGA-3′; U6: F: 5′-CTCGCTTCGGCAGCACA-3′, R: 5′-AACGCTTCACGAATTTGCGT-3′.

### 2.3. Western Blotting

The OSCC cells were lysed in RIPA buffer with protein inhibitors and the protein content was quantified using a BCA kit. The proteins were separated on 10% SDS polyacrylamide gels and electrically transferred to PVDF membranes (Millipore). The membranes were blocked with no-fat milk before incubation with the primary and corresponding secondary antibodies. Antibodies used in this study were as follows: anti-HSPD1 (CST, ^#^ 12165S, 1 : 1000), GAPDH (CST, ^#^ 5174S, 1 : 1000).

### 2.4. Ethynyl‐2‐Deoxyuridine (EdU) Incorporation Assay

The incorporation of EdU into DNA was applied to detect cells undergoing DNA replication. After cell fixation and permeabilization, cells were incubated with 50 *μ*M EdU solution for 3 hours and stained with 1 *μ*g/m DAPI for 10 mins. Fluorescence microscopy was used to visualize and count the EdU-positive cells.

### 2.5. Transwell Assay

Transwell assays were performed using Transwell chambers (Corning, USA). Infected cells were added to the upper chamber, and a culture medium with 20% FBS was added to the lower chamber. The migrated and invaded cells were fixed with 4% paraformaldehyde and stained with 0.5% crystal violet, then visualised with an Olympus microscope.

### 2.6. Detection of Warburg Effect OSCC Cells

The Warburg effects relative to glucose uptake, lactate production and ATP consumption were evaluated to assess the effect of the circMAT2B/miR-942-5p/HSPD1 axis in OSCC cells using commercial kits (Glucose uptake (#K924), lactate production (#K627), ATP generation (#K345), BioVision, USA) according to previous studies.

### 2.7. RNA Pull-Down

The biotinylated RNA pull-down assay was conducted using MyOne™ Dynabeads® Streptavidin C1 beads. The beads were incubated with cell lysates for 60 mins at 4°C for preclearance. The RNA-probes were synthesized and commercially procured by Tsingke Biotech (Beijing, China). Subsequently, probes were incubated with beads for 10 min at room temperature for immobilization, and then incubated with the cell lysates for 12 hours overnight. The beads were magnetically separated and washed five times before qRT-PCR.

### 2.8. Luciferase Reporter Assay

The luciferase assay was performed using the dual-luciferase reporter system psiCHECKTM (Fisher Scientific). Cells (4 × 10^4^ cells per well) were cultured in 24-well plates overnight and transfected with Wide Type (WT) or Mutant Type (MUT) vectors together with the plasmid for Renilla luciferase expression. Lipofectamine 2000 (Invitrogen) was used to perform transfections. After 24 hours, the cells were lysed, and their luciferase activities were measured using the dual-luciferase reporter assay system (Promega).

### 2.9. Statistical Analysis

The data were analysed using SPSS 20.0 (IBM, USA) and presented as the mean ± standard deviation (mean ± SD). One-way ANOVA test or Student's *t*-test was used to determine the statistical significance and *P* < 0.05 was considered statistically significant. All experiments were repeated three times.

## 3. Results

### 3.1. Characterization of circMAT2B in OSCC

**Figure 1:**
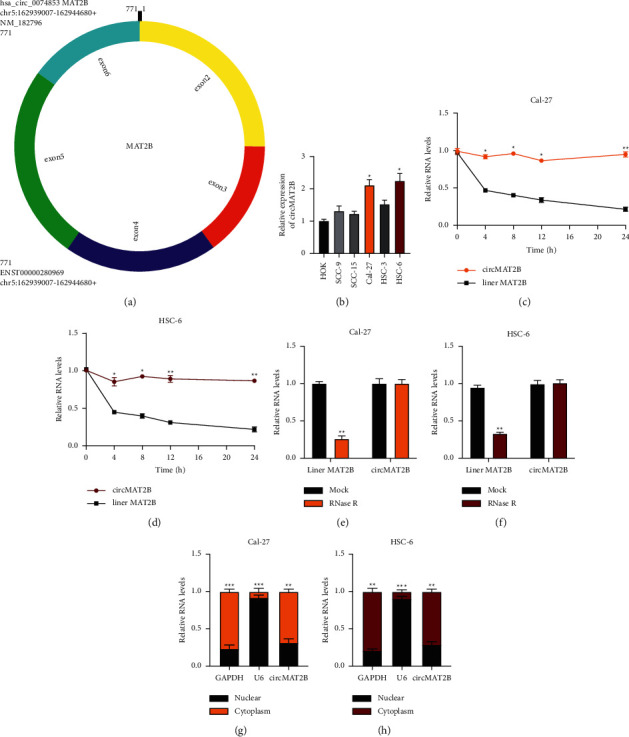
Characterization of circMAT2B in OSCC. (a) The exonic information of circMAT2B. (b) Relative expression of circMAT2B in HOK and OSCC cell lines was measured by qRT-PCR. (c) and (d) Cal-27 (d) and HSC-6 (d) cells were treated with actinomycin D and relative RNA levels were detected by qRT-PCR at the indicated time. (e) and (f) Total RNAs from Cal-27 (e) and HSC-6 (f) cells were treated with RNase R or mock and relative RNA levels were detected by qRT-PCR. G-H: Cellular RNA fractionation was conducted to assess circMAT2B distribution in Cal-27 (g) or HSC-6 (h). GAPDH or U6 was used as an internal control. Each experiment was performed three times. Data are presented as mean ± SD. ^*∗*^*P* < 0.05, ^*∗∗*^*P* < 0.01, ^*∗∗∗*^*P* < 0.001.

The expression of circMAT2B (Figure 1(a)) in OSCC cell lines (HOK, SCC-9, SCC-15, Cal-27, HSC-3, and HSC-6) was generally elevated, especially in Cal-27 and HSC-6 cells (Figure 1(b)). Next, Actinomycin D and RNase R were applied to evaluate the stability of circMAT2B in OSCC cells, showing that circMAT2B was more stable than its liner form in Cal-27 and HSC-6 cells (Figures 1(c) and 1(d)) and more resistant to Rnase R digestion than its liner form in Cal-27 and HSC-6 cells (Figures 1(e) and 1(f)). Subsequently, circMAT2B was found to be mainly distributed in OSCC cell cytoplasm (Figures 1(g) and 1(h)), indicating its potential for biological function. It was hypothesized that circMAT2B is involved in OSCC progression.

### 3.2. CircMAT2B Knockdown Inhibits OSCC Tumorigenesis and the Warburg Effect

**Figure 2:**
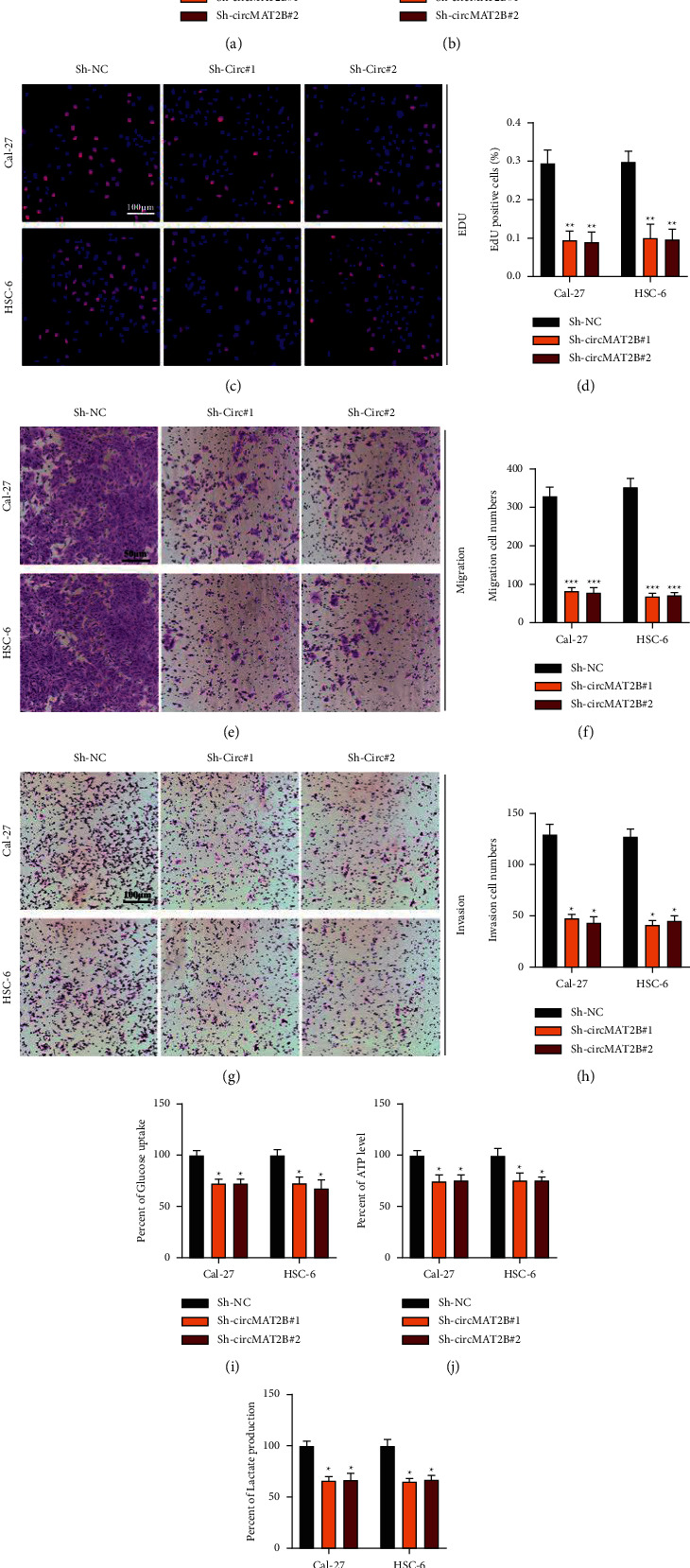
CircMAT2B knockdown inhibits OSCC tumorigenesis and the Warburg effect. (a) and (b) OSCC cells Cal-27 and HSC-6 were infected with Sh-NC, Sh-circMAT2B#1, and Sh-circMAT2B#2, respectively. CircMAT2B (a) and MAT2B (b) expression was measured by qRT-PCR. (c) and (d) CircMAT2B knockdown or normal control Cal-27 or HSC-6 cells were subjected to EdU assay for proliferation ability detection (c) and analysis (d). (e) and (f) transwell migration assay was performed to evaluate migration level of circMAT2B knockdown or normal control Cal-27 or HSC-6 cells (e) and (f). (g) and (h) transwell invasion assay was performed to evaluate the invasion of circMAT2B knockdown or normal control Cal-27 or HSC-6 cells (g) and (h). (i)–(k) warburg effect relative glucose uptake (i), lactate production (j), and ATP levels (k) were detected. Each experiment was performed three times. Data are presented as mean ± SD. ^*∗*^*P* < 0.05, ^*∗∗*^*P* < 0.01, ^*∗∗∗*^*P* < 0.001.

To investigate whether circMAT2B plays a biological function in OSCC development, circMAT2B knockdown cell models were created (Figures 2(a) and 2(b)). As evidenced by EdU and Tranwell assays, circMAT2B significantly inhibited OSCC cell proliferation (Figures 2(c) and 2(d)), migration (Figures 2(e) and 2(f)), and invasion (Figures 2(g) and 2(h)). There is emerging evidence that circRNAs exert their biological function in cancer progression via modulating the Warburg effect, [26, 27]. We examined the Warburg effect relative to glucose uptake, lactate production, and ATP level showing that circMAT2B knockdown suppressed the Warburg effects (Figures 2(i)–2(k)).

### 3.3. CircMAT2B Expression Is Transcriptionally Regulated by TEAD1

**Figure 3:**
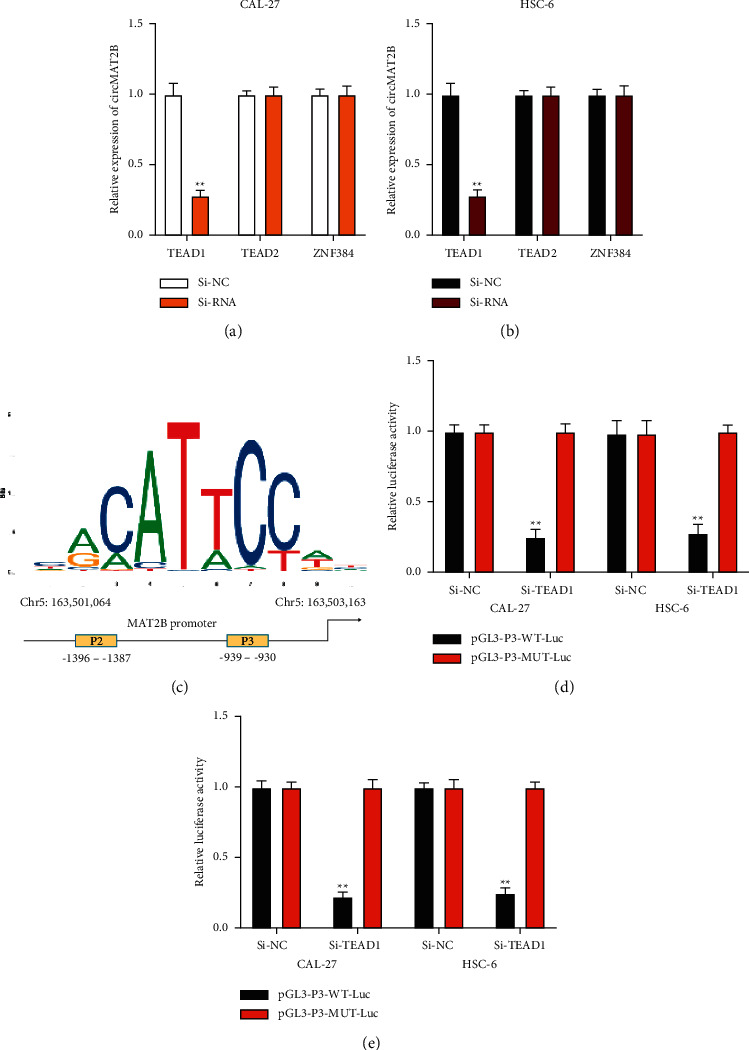
CircMAT2B expression is transcriptionally regulated by TEAD1. Upstream transcriptional regulators of circMAT2B were predicted using the JASPAR dataset with a relative profile score threshold over 95%. (a) and (b) Relative expression of circMAT2B in Cal-27 and HSC-6 cells on si-TEAD1, si-TEAD2, si-ZNF384, and normal control (si-NC) transfection was measured by qRT-PCR. (c) The predicted binding sites between TEAD1 and MAT2B promoter regions P2 and P3. (d) and (e) Dual-luciferase reporter gene assay was applied to evaluate the interaction between TEAD1 and P2/P3 region of MAT2B promoter in Cal-27 and HSC-6 cells. Each experiment was performed three times. Data are presented as mean ± SD. ^*∗*^*P* < 0.05, ^*∗∗*^*P* < 0.01.

The JASPAR dataset (https://jaspar.genereg.net/) was used to investigate whether circMAT2B expression in OSCC cells is transcriptionally regulated. Three potential transcriptional regulators were identified (TEAD1, TEAD2, and ZNF384) and as shown in Figures 3(a) and 3(b), the expression of circMAT2B was significantly decreased upon si-TEAD1 infection in Cal-27 and HSC-6 cells indicating that TEAD1 might regulate circMAT2B expression. The predicted binding sites of TEAD1 on MAT2B promoter sequences are presented in Figure 3(c). Subsequently, the interaction between TEAD1 and MAT2B promoter regions P2 and P3 was confirmed by conducting luciferase reporter gene assays in Cal-27 and HSC-6 cells as indicated (Figures 3(d) and 3(e)).

### 3.4. CircMAT2B Sponges to miR-942-5p

**Figure 4:**
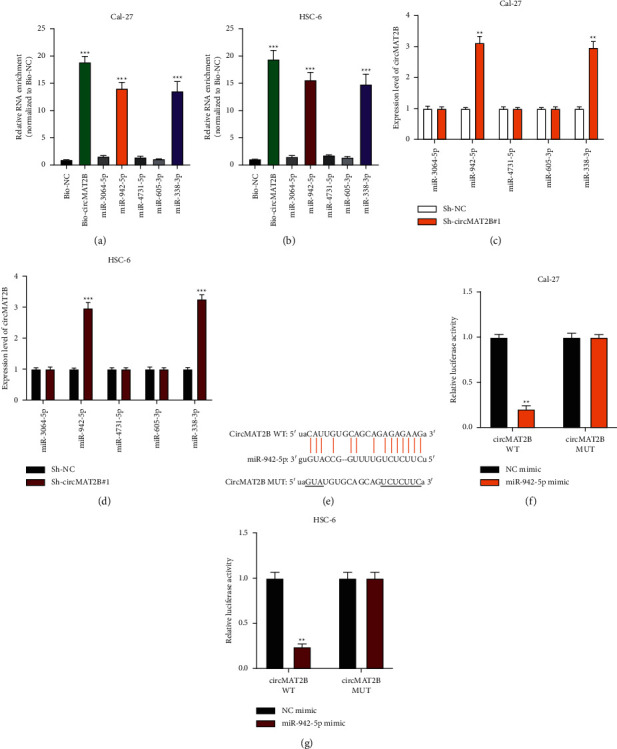
CircMAT2B sponges to miR-942-5p. a-b: biotinylated NC probes and circMAT2B probes were transfected into Cal-27 (a) and HSC-6 (b) cell and putative miRNA target expression in pull-down bounds was measured by qRT-PCR. C-D: Sh-NC or Sh-circMAT2B#1 was infected into Cal-27 (c) or HSC-6 (d) cells and the relative expression of putative miRNA targets was measured by qRT-PCR. (e): bioinformatic prediction of circMAT2B-miR-942-5p binding. (f) and (g): a luciferase reporter gene assay was conducted to confirm the interaction between circMAT2B and miR-942-5p. Each experiment was performed three times. Data are presented as mean ± SD. ^*∗*^*P* < 0.05, ^*∗∗*^*P* < 0.01, ^*∗∗∗*^*P* < 0.001.

The underlying mechanisms of circMAT2B in OSCC progression were investigated by bioinformatics analysis (Starbase: https://starbase.sysu.edu.cn/index.php, CLIP Data: strict stringency (≥5), and Class defined as 8 mer). Four putative miRNA targets (miR-3064-5p, miR-942-5p, miR-4731-5p, and miR-605-3p) of circMAT2B were found. Biotinylated RNA pull-down assay results suggested that miR-942-5p might be a downstream factor of circMAT2B in OSCC cells (Figures 4(a) and 4(b)). MiR-338-3p, which is a validated target for circMAT2B [23], was used as a positive control. Furthermore, circMAT2B knockdown reduced miR-942-5p expression in OSCC cells (Figures 4(c) and 4(d)). The bioinformatic predicted binding sites between circMAT2B and miR-942-5p were located as indicated in Figure 4(e). The dual-luciferase reporter gene assay confirmed the interaction between circMAT2B and miR-942-5p in Cal-27 and HSC-6 cells (Figures 4(f) and 4(g)). These results suggest that circMAT2B sponges to miR-942-5p and negatively regulates miR-942-5p expression in OSCC cells. To verify the biological role of miR-942-5p in circMAT2B modulated OSCC cellular behaviors, we conducted rescue experiments, including EdU, Transwell migration assay, Transwell invasion assay, and Warburg effects detection. The results (presented in supplementary Figure S) suggest that downregulation of miR-492-5p reversed the inhibitory impacts of circMAT2B knockdown on OSCC cell proliferation, migration, invasion, and the Warburg effects.

### 3.5. MiR-942-5p Overexpression Suppresses Tumorigenesis and the Warburg Effect in OSCC

**Figure 5:**
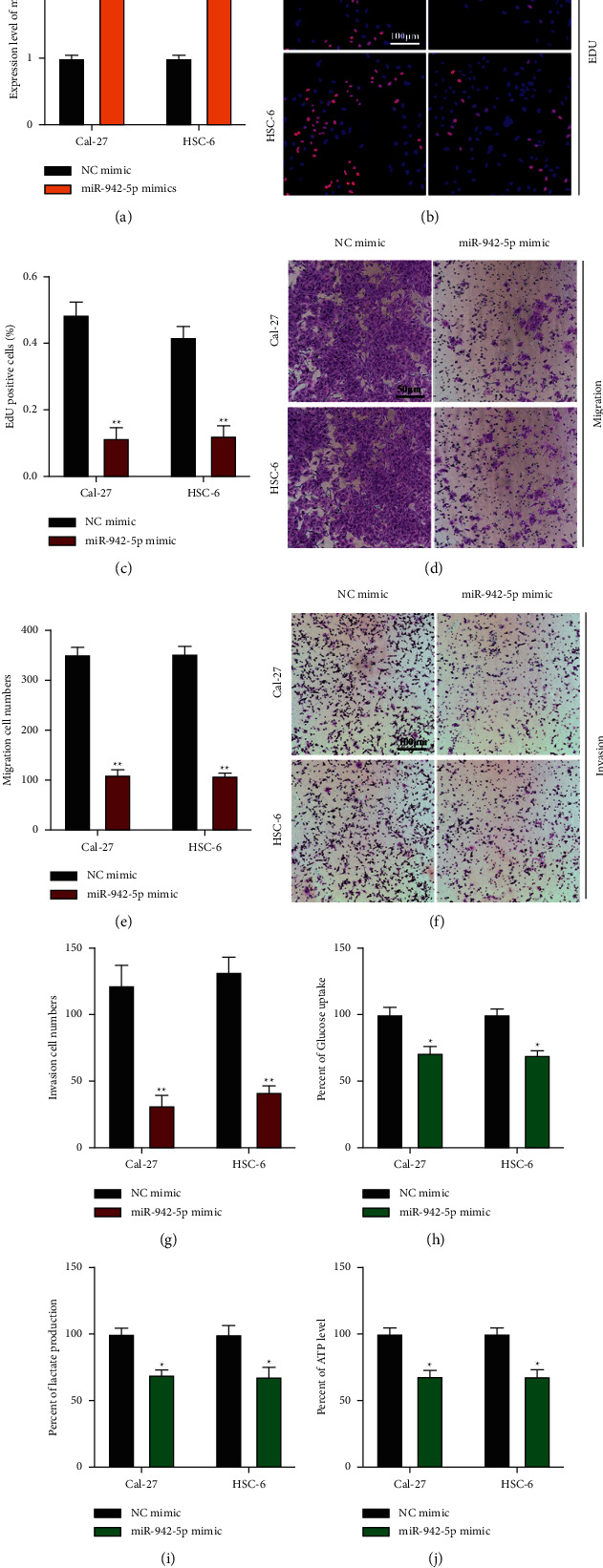
MiR-942-5p overexpression suppresses tumorigenesis and the Warburg effect in OSCC. (a): MiR-942-5p overexpression cell models were generated by infecting miR-942-5p mimic or its normal control into Cal-27 or HSC-6 cells and the expression of miR-942-5p was measured by qRT-PCR. (b) and (c): cell proliferation was detected by EdU assay (b) and (c). (d) and (e): cell migration was assessed by transwell migration assay (d) and (e). (f) and (g): cell invasion was measured by the transwell invasion assay (f) and (g). (h)–(j): The warburg effect relative glucose uptake (h), lactate production (i), and ATP levels (j) in OSCC cells. Each experiment was performed three times. Data are presented as mean ± SD. ^*∗*^*P* < 0.05, ^*∗∗*^*P* < 0.01, ^*∗∗∗*^*P* < 0.001.

MiR-942-5p overexpression cell models were constructed by infecting miR-942-5p mimic and a control mimic into Cal-27 and HSC-6 cells (Figure 5(a)). As evidenced by EdU and Transwell assays, miR-942-5p overexpression inhibited OSCC cell proliferation (Figures 5(b) and 5(c)), migration (Figures 5(d) and 5(e)), and invasion (Figures 5(f) and 5(g)). Moreover, miR-942-5p overexpression suppressed the relative glucose uptake (Figure 5(h)), lactate production (Figure 5(i)), and ATP level (Figure 5(j)) in OSCC cells. In summary, the inhibitive effects of miR-942-5p in tumorigenesis and the Warburg effect in OSCC cells were elucidated.

### 3.6. MiR-942-5p Directly Targets HSPD1

**Figure 6:**
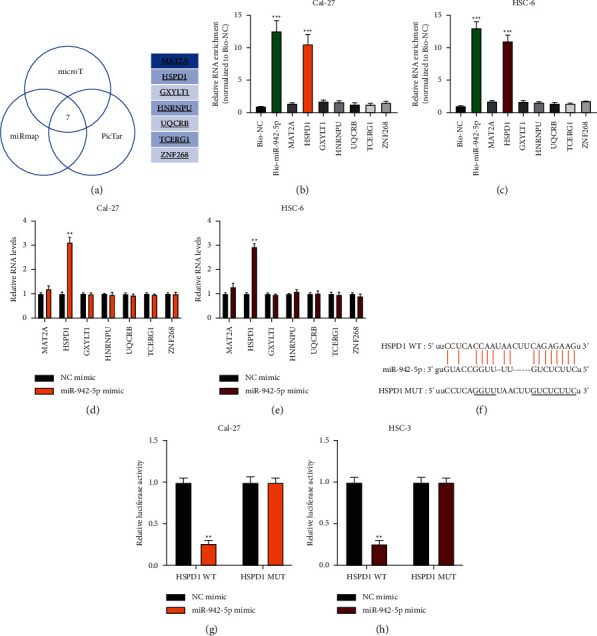
MiR-942-5p directly targets HSPD1. (a): potential mRNA targets for HSPD1 predicted by bioinformatics analysis. (b) and (c): biotinylated NC probes and miR-942-5p probes were transfected into Cal-27 (b) and HSC-6 (c) cells and the putative miRNA target expression in pull-down bounds was measured by qRT-PCR. D-E: NC mimic or miR-942-5p mimic was infected into Cal-27 (d) or HSC-6 (e) cells and the relative expression of putative mRNA targets was measured by qRT-PCR. (f): bioinformatic prediction of the HSPD1-miR-942-5p binding. (g) and (h): A luciferase reporter gene assay was conducted to confirm the interaction between HSPD1 and miR-942-5p. Each experiment was performed three times. Data are presented as mean ± SD. ^*∗*^*P* < 0.05, ^*∗∗*^*P* < 0.01, ^*∗∗∗*^*P* < 0.001.

The microT (https://diana.imis.athena-innovation.gr/), miRmap (https://mirmap.ezlab.org/), and PicTar (https://pictar.mdc-berlin.de/) datasets were used to predict the downstream targets for miR-942-5p and identified seven mRNA candidates (Figure 6(a)). Biotinylated RNA pull-down assay results indicated that miR-942-5p might target HSPD1 in OSCC cells (Figures 6(b) and 6(c)), as HSPD1 expression in OSCC cells could be suppressed by miR-942-5p overexpression (Figures 6(d) and 6(e)). The predicted binding sites between HSPD1 and miR-942-5p are shown in Figure 6(f). The dual-luciferase reporter gene assay confirmed that miR-942-5p directly targets HSPD1 in OSCC cells (Figures 6(g) and 6(h)).

### 3.7. CircMAT2B Accelerates OSCC Progression through the miR-942-5p/HSPD1 Axis

**Figure 7:**
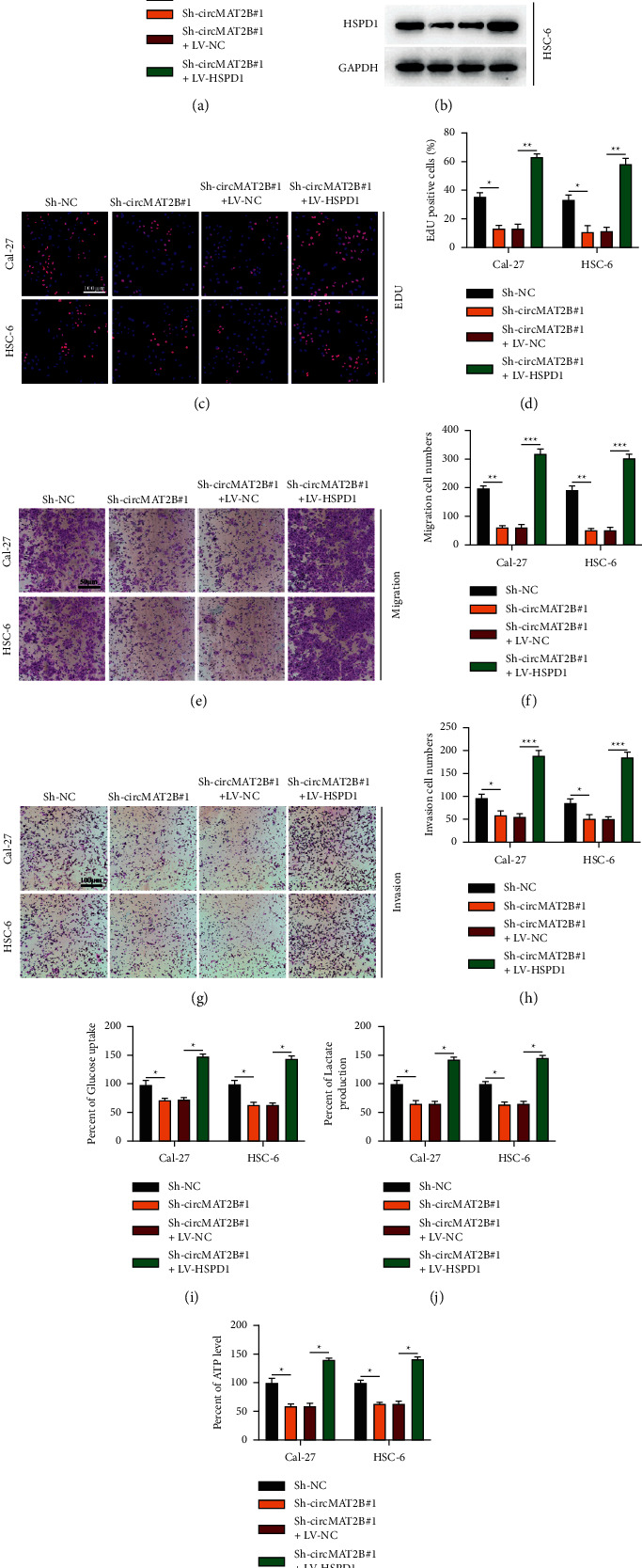
CircMAT2B accelerates OSCC progression through the miR-942-5p/HSPD1 axis. (a) and (b): cell models were constructed by transfecting Sh-NC, Sh-circMAT2B#1, Sh-circMAT2B#1 + LV-NC, Sh-circMAT2B#1 + LV-HSPD1 into Cal-27 or HSC-6 cells as indicated, transfection efficiency was assessed by qRT-PCR (a) and Western blotting (b). (c) and (d): Cell proliferation was detected by the EdU assay (c) and (d). (e) and (f) cell migration was assessed by the transwell migration assay (e) and (f). (g) and (h) cell invasion was measured by the transwell invasion assay (f) and (h). (i)–(k) Warburg effect relative glucose uptake (i), lactate production (j), and ATP levels (k) in OSCC cells. Each experiment was performed three times. Data are presented as mean ± SD. ^*∗*^*P* < 0.05, ^*∗∗*^*P* < 0.01, ^*∗∗∗*^*P* < 0.001.

Cell models were generated as indicated to exploit the circMAT2B/miR-942-5p/HSPD1 axis in OSCC progression (Figures 7(a) and 7(b)). CircMAT2B knockdown suppressed the proliferation (Figures 7(c) and 7(d)), migration (Figures 7(e) and 7(f)), and invasion (Figures 7(g) and 7(h)) of OSCC cells, while those phenomena could be rescued by HSPD1 overexpression. Moreover, the inhibitive effect of circMAT2B knockdown on Warburg effect relative phenomena in OSCC cells was also reversed by HSPD1 overexpression (Figures 7(i)–7(k)). Overall, our study elucidated that circMAT2B modulates HSPD1 expression to promote OSCC progression through sponging miR-942-5p.

## 4. Discussion

Head and neck squamous cell carcinoma (HNSCC) accounts for more than 800,000 new cases and causes about 450,000 cancer-related deaths in 2018. Of these, OSCC accounts for approximately 95% of HNSCC and is responsible for 350,000 new cases and 170,000 cancer-related deaths worldwide in 2018 [28]. OSCC generally originates in the tongue, gingiva, mouth floor, lips, alveolar ridge, buccal mucosa, hard palate, and retromolar trigone, with surgical resection with or without lymph nodes removal being the primary clinical treatment. However, the high recurrence and metastasis formation of OSCC lead to poor survival and life quality of OSCC patients [29, 30]. Therefore, novel diagnostic strategies and therapeutic targets for OSCC are necessary.

With the advent of high-through putting sequencing, multiple circRNAs in various cancers have been investigated and verified, including OSCC. In the current study, we investigated circMAT2B function in OSCC progression. CircMAT2B was highly expressed in OSCC tumor tissues and mainly expressed in the cell cytoplasm. The downregulation of circMAT2B in OSCC cells inhibited cell proliferation, migration, invasion and the Warburg effect.

The Warburg effect is an important phenomenon of aerobic glycolysis which maintains tumor cell proliferation and aggressive characteristics [31]. The Warburg effect-related limiting enzymes including hexokinase-2 (HK2), phosphofructokinase-2 (PFK2), pyruvate kinase-2 (PKM2), pyruvate dehydrogenase kinase-1 (PDK1), and lactate dehydrogenase (LDH), modulate glycolysis rate, lactate production, and ATP level in tumor cells and influence tumor cell biosynthesis, the immune response, and progression [32–34]. Herein, we found that cirMAT2B knockdown inhibited the Warburg effect relative phenomenon in OSCC cells, suggesting an oncogenic role of circMAT2B in OSCC development.

Subsequently, circMAT2B expression in OSCC could be regulated by transcriptional regular TEAD1. Furthermore, by conducting bioinformatic analysis, RNA pull-down, and luciferase reporter gene assays, a novel circMAT2B/miR-942-5p/HSPD1 axis was identified in OSCC cells. A previous study reported that miR-942-5p expression is upregulated in OSCC tumor tissues and contributes to OSCC progression [35], whereas the role of HSPD1 in OSCC progression is still unclear. The present study found that circMAT2B upregulated HSPD1 expression to modulate OSCC cell proliferation, migration, invasion, and Warburg effect level through sponging miR-942-5p.

## 5. Conclusion

Our study has partially demonstrated a novel circMAT2B/miR-942-5p/HSPD1 axis in OSCC progression and that TEAD1 transcriptionally regulates circMAT2B expression in OSCC cells. The upstream and downstream molecular mechanisms of circMAT2B in OSCC cells have been explored, providing a new diagnostic or therapeutic target for OSCC.

## Data Availability

The data supporting the conclusions of this article will be made available by the authors, without undue reservation.

## Supplementary Materials

Supplementary Materials Supplementary Figure S MiR-492-5p reversed the inhibitory effects of circMAT2B on OSCC cellular process. A-B: cell models were constructed by transfecting Sh-NC, Sh-circMAT2B#1, Sh-circMAT2B#1 + si-NC, and Sh-circMAT2B#1 + si-miR-492-5p into Cal-27 or HSC-6 cells as indicated, transfection efficiency was assessed by qRT-PCR (A) and Western blot (B). C-D: cell proliferation level was detected by EDU assay (C) and results were statistically analyzed (D). E-F: cell migration level was assessed by Transwwell migration assay (E) and results were calculated (F). G-H: cell invasion level was measured by Transwwell invasion assay (F) and results were analyzed (H). I-K: Warburg effect relative glucose uptake (I), lactate production (J), and ATP levels (K) in OSCC cells were detected. Each experiment was performed three times. Data is presented as the Mean ± SD. ^*∗*^*P* < 0.05, ^*∗∗*^*P* < 0.01, ^*∗∗∗*^*P* < 0.001.
