# Supplementary material for: CircMAT2B Induced by TEAD1 Aggravates the Warburg Effect and Tumorigenesis of Oral Squamous Cell Carcinoma through the miR-942-5p/HSPD1 Axis
Source: J Oncol. 2022 Aug 16;2022:7574458. doi: 10.1155/2022/7574458 (PMC9398833; doi:10.1155/2022/7574458)
Supplement: Supplementary Materials — Supplementary Figure S MiR-492-5p reversed the inhibitory effects of circMAT2B on OSCC cellular process. A-B: cell models were constructed by transfecting Sh-NC, Sh-circMAT2B#1, Sh-circMAT2B#1 + si-NC, and Sh-circMAT2B#1 + si-miR-492-5p into Cal-27 or HSC-6 cells as indicated, transfection efficiency was assessed by qRT-PCR (A) and Western blot (B). C-D: cell proliferation level was detected by EDU assay (C) and results were statistically analyzed (D). E-F: cell migration level was assessed by Transwwell migration assay (E) and results were calculated (F). G-H: cell invasion level was measured by Transwwell invasion assay (F) and results were analyzed (H). I-K: Warburg effect relative glucose uptake (I), lactate production (J), and ATP levels (K) in OSCC cells were detected. Each experiment was performed three times. Data is presented as the Mean ± SD. ∗P < 0.05, ∗∗P < 0.01, ∗∗∗P < 0.001. [file 7574458.f1.docx]

**Supplementary Figure S**


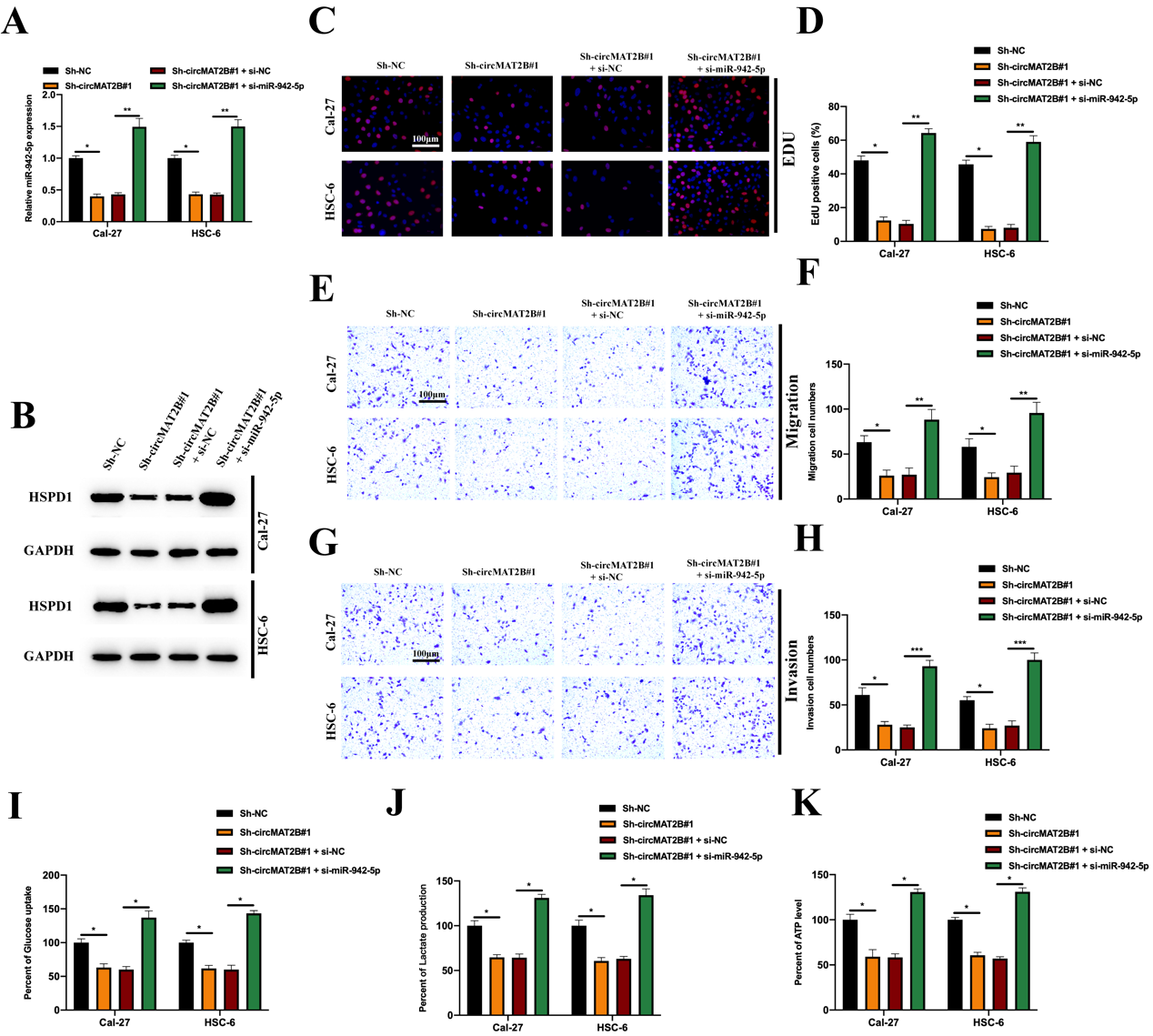


**MiR-492-5p reversed the inhibitory effects of circMAT2B on OSCC cellular process.** A-B: Cell models were constructed by transfecting Sh-NC, Sh-circMAT2B#1, Sh-circMAT2B#1 + si-NC, Sh-circMAT2B#1 + si-miR-492-5p into Cal-27 or HSC-6 cells as indicated, transfection efficiency was assessed by qRT-PCR (A) and western blot (B). C-D: Cell proliferation level was detected by EDU assay (C), results were statistically analyzed (D). E-F: Cell migration level was assessed by Transwwell migration assay (E), results were calculated (F). G-H: Cell invasion level was measured by Transwwell invasion assay (F), results were analyzed (H). I-K: Warburg effect relative glucose uptake (I), Lactate production (J), and ATP levels (K) in OSCC cells were detected. Each experiment was performed three times. Data was presented as Mean ± SD. **P* < 0.05, ***P* < 0.01, ****P* < 0.001.
